# From fruit waste to foods via fermentation

**DOI:** 10.1016/j.crfs.2026.101549

**Published:** 2026-08-27

**Authors:** Ines Calvete-Torre, Samuel Breselge, John Leech, Harsh Mathur, Paul D. Cotter

**Affiliations:** a Food Biosciences Department, Teagasc Food Research Centre, Moorepark, Fermoy, Co. Cork, P61 C996, Ireland; b Functional Beverage Technology, TUM School of Life Sciences, Technical University of Munich, Weihenstephan, Gregor-Mendel-Straße 4, Freising, 85354, Germany; c APC Microbiome Ireland, University College Cork, Cork, Ireland; d VistaMilk, Moorepark, Fermoy, Co. Cork, P61 C996, Ireland

**Keywords:** Fruit by-products, Microbial fermentation, Bioactive compounds, Plant-based matrices, Sustainable valorisation

## Abstract

The increasing generation of fruit waste represents a major environmental and economic challenge for the agri-food sector, while also offering significant opportunities for sustainable valorisation within the framework of the circular economy. Fruits rejected for cosmetic reasons, together with processing by-products, are rich sources of dietary fibre, sugars and bioactive compounds that can be converted into value-added products. This review aims to provide a comprehensive overview of microbial fermentation as a sustainable strategy for fruit waste valorisation, with particular emphasis on the role of lactic acid bacteria, acetic acid bacteria, yeasts and microbial consortia in improving the nutritional, technological and functional properties of fruit-derived substrates.

Current evidence demonstrates that microbial fermentation not only preserves fruit biomass but also enhances its value through the degradation of complex carbohydrates, the biotransformation of phenolic compounds, the production of bioactive metabolites and the generation of functional ingredients. This review further discusses the contribution of viable microorganisms and postbiotic components to product functionality, as well as the importance of microbial viability, processing conditions and storage stability for the development of safe and functional fermented foods. In addition, it highlights recent advances in the use of microbial consortia and identifies the mechanisms governing microorganism–plant matrix interactions as a key area requiring further investigation.

Overall, the available literature supports fermentation as a versatile and effective strategy for transforming fruit waste into high-value food products. By integrating current knowledge on microbial metabolism, functionality and processing challenges, this review identifies the main scientific and technological gaps and outlines future research priorities, including microbial consortium design, process scale-up, regulatory considerations and life cycle assessment, to facilitate the industrial implementation of sustainable fruit waste biorefineries.

## Highlights

• Fruit residues are promising substrates for microbial fermentation.
• Fermentation enhances the nutritional and sensory value of fruit residues.
• Microbial bioprocesses generate functional ingredients from fruit waste.
• Future progress requires scalable, safe and sustainable bioprocesses.

## Graphical abstract

**Figure:**
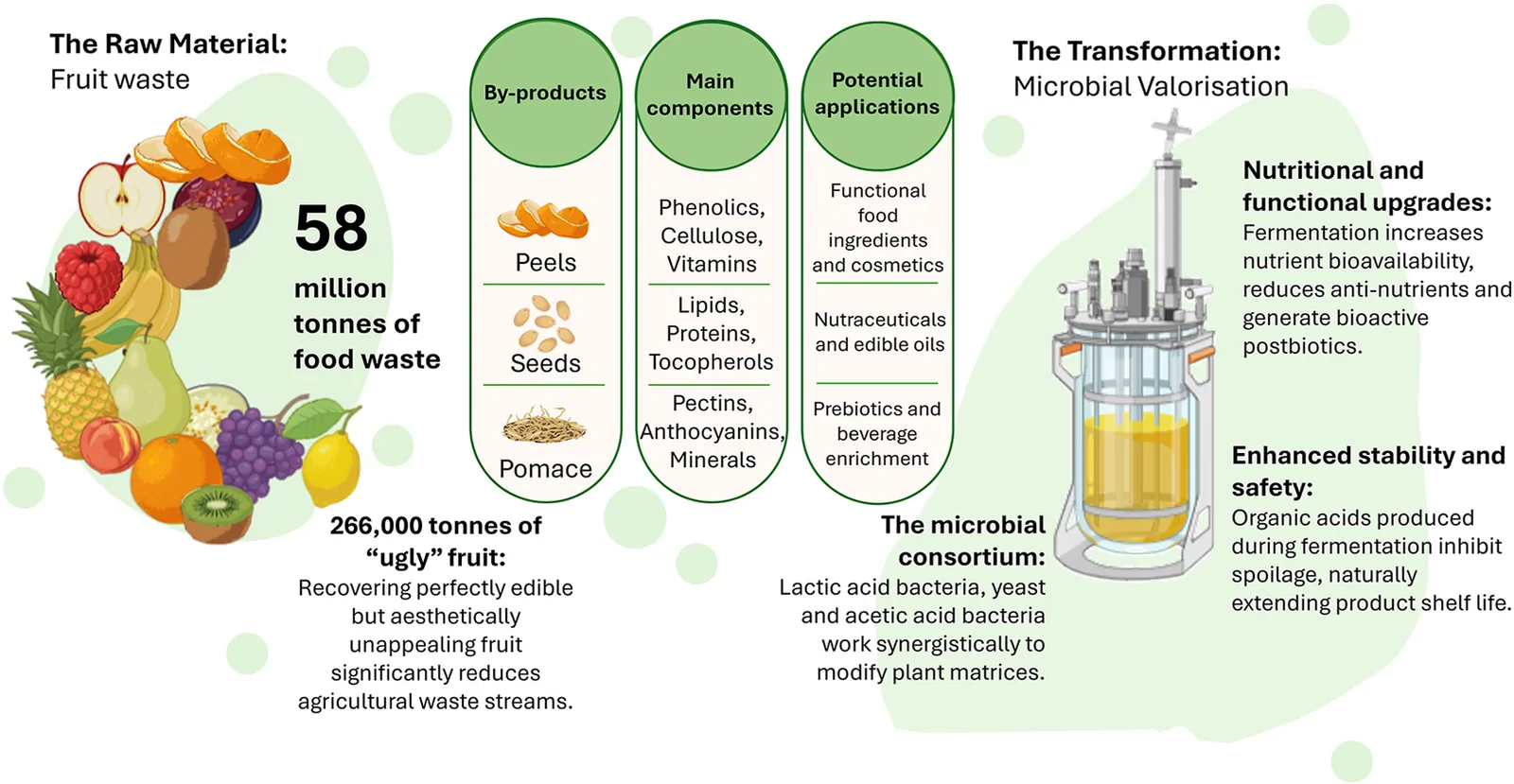

## 1 Introduction

Food waste is one of the defining sustainability challenges facing the modern agri-food sector. In the European Union alone, more than 58 million tonnes are generated annually, with consequences that are simultaneously economic, in the form of lost revenue for farmers and food businesses; social, in that edible calories are discarded against a background of persistent hunger and malnutrition; and environmental, with the EU food system accounting for approximately 16% of total greenhouse gas emissions (Soares et al., 2025).

A large proportion of agri-food waste comes from fruit production and processing. In 2020, 1% of the total waste (2,153,950,000 tonnes) produced in the EU was from the agricultural sector (Meena et al., 2023). The growing global population and changing dietary habits have considerably increased the demand for fruit, leading to greater production and processing volumes in recent years (Kaushik et al., 2025). Food waste is generated at every stage of the supply chain, from pre-harvest (10%) and processing losses (19%) to those associated with distribution (8%), retail, and final consumption in households (53% or 69 kg per inhabitant) and restaurants (11%) (Makhal et al., 2021). Against this backdrop, the Sustainable Development Goals have driven a rapid expansion in research into agri-food waste management.

Fruit waste primarily consists of fruit that does not meet optimal standards for customers (including undesirable shapes or colours) and fruit waste generated after processing (Nirmal et al., 2023). The waste generated during fruit processing depends on the final product being made, but the most common are peels, seeds, pulp, pomace, and bagasse (Sharma et al., 2025). This waste is commonly disposed of in landfills with the high nutrient and organic matter content leading to soil and water pollution, as well as the production of greenhouse gases. Fruit waste can be used as animal feed; however, it is important to note its use must be controlled to avoid nutritional imbalances and possible health risks for the animals (Sharma et al., 2025; Kavitha Shree et al., 2025). Developing sustainable strategies that reduce these losses while converting them into value-added products and contributing to the circular economy therefore remains a considerable challenge.

In accordance with the principles of the circular economy, fruit waste is increasingly regarded as a valuable bioresource rather than a waste product. Its richness in dietary fibre, sugars, organic acids, vitamins, minerals and phytochemicals makes it an attractive feedstock for a wide range of industrial applications, transforming an environmental challenge into a biotechnological opportunity (Kavitha Shree et al., 2025). Fruit waste is rich in bioactive compounds, defined as “essential and non-essential compounds (e.g., vitamins or polyphenols) that occur in nature, are part of the food chain, and can be shown to have an effect on human health” (Biesalski et al., 2009). Fruit waste can be utilized through chemical and biological processes, allowing the extraction of bioactive compounds, such as phenols, flavonoids, carbohydrates, lipids, and minerals and their use as natural alternatives to chemically synthesized compounds (Deng et al., 2012). This offers great potential for applications in both the food and pharmaceutical sectors and would enable the production of functional foods with multiple health-promoting properties: antioxidant, anti-inflammatory, and cytoprotective activities (Caponio et al., 2023; Monteiro et al., 2021). Realising this potential is not, however, straightforward. Conventional recovery by solvent extraction is frequently energy-intensive and generates its own waste streams, and it leaves the depleted matrix unused. Moreover, the physiological relevance of the compounds recovered depends on their digestive stability, their bioaccessibility, and their interaction with the gut microbiota, parameters that remain incompletely characterised for many fruit-derived bioactives (Lippolis et al., 2023).

Regarding the food sector, and in response to consumer trends with a growing interest in functional foods that improve health and reduce the risk of disease, one possible solution for fruit waste is the production of fermented beverages (Marsh et al., 2014). Fermentation also represents another valuable approach to obtain bioactive compounds. Microorganisms possess enzymes that can transform food matrices, improving the bioavailability of existing nutrients or transforming them into new bioactive compounds. Fermented foods have traditionally been used for preservation purposes, but in some cases, they have also demonstrated health-promoting potential (Park and Mannaa, 2025; Li et al., 2024).

Traditional fermented foods are commonly produced through the activity of complex microbial communities, which can include microorganisms with potential probiotic properties (Park and Mannaa, 2025; Li et al., 2024). These microorganisms are typically lactic acid bacteria (LAB), acetic acid bacteria (AAB), yeasts, and other microorganisms, and can be found in fermented foods such as yogurt, kefir, cheese, kimchi, sauerkraut, tempeh, and miso. They can also be found naturally on the surface of fruits, making them key microorganisms for carrying out fermentation processes (Li et al., 2024). The combined use of these microorganisms can enable the development of novel fermented foods and the valorisation of fruit residues, thus supporting both functional food innovation and circular economy strategies (Li et al., 2024; Zamani et al., 2026). Other mechanisms, besides the possible probiotic capacity of viable microorganisms, such as physiological effects that can also come from inanimate microorganisms and/or their structural components known as postbiotics may also contribute to health (Salminen et al., 2021).

In addition to the potential probiotic or postbiotic contribution of fermented foods, microorganisms can enzymatically transform complex carbohydrates, resulting in structural and biochemical changes in the food matrix. These transformations promote nutrient release, modify phenolic profiles and generate a variety of bioactive compounds that contribute to the functional properties of fermented products. Compounds such as short-chain fatty acids, bioactive peptides, exopolysaccharides and microbially transformed phytochemicals have shown the potential to modulate immune responses, improve intestinal barrier function and influence host metabolic pathways in *in vitro* and in animal studies. However, evidence from human intervention studies remains limited, and further clinical research is required to confirm these effects in the context of fermented fruit products (Mukherjee et al., 2024). The production of organic acids contributes to flavour development and improves product stability by creating an acidic environment that preserves proteins and phenolic compounds while inhibiting the growth of spoilage microorganisms (Yang et al., 2024). At the same time, fermentation improves the bioavailability of nutrients, can increase vitamin levels, reduce antinutritional factors such as phytates and tannins, decrease potential allergenicity through microbial proteolysis, and partially degrade fermentable carbohydrates, thus contributing to better gastrointestinal tolerance (Marco et al., 2017).

Despite these advances, the available evidence remains fragmented. While numerous studies have described individual aspects of fruit waste fermentation, relatively few reviews have integrated the biochemical characteristics of fruit residues, microbial metabolism, product functionality, microbial viability and industrial applicability into a single framework. Existing reviews have therefore tended to address these topics separately, with limited critical comparison of fermentation strategies, microbial systems and reported outcomes. The present review addresses this gap through four main objectives: (i) to characterise the biochemical properties of the principal fruit residue streams that determine their suitability as fermentation substrates; (ii) to critically evaluate the roles of LAB, AAB, yeasts and defined microbial consortia in transforming these matrices; (iii) to analyse the enzymatic and metabolic mechanisms by which fermentation modifies bioactive profiles, including carbohydrate degradation, phenolic transformation and *de novo* metabolite generation; and (iv) to examine the determinants of microbial viability, product stability and safety, encompassing processing conditions, storage and microbial inactivation strategies.

The distinctive contribution of this review lies in its integrative and critical perspective. Rather than providing an exhaustive catalogue of published studies, we compare the available evidence across different substrates, fermentation systems and microbial groups, highlighting methodological limitations, inconsistencies and current knowledge gaps. Particular attention is given to the mechanisms underlying functional improvements, the role of microbial viability and postbiotics, and the translational barriers that separate laboratory findings from commercial implementation. These include the compositional variability of fruit residues, seasonality and supply chain logistics, unintended alcohol production, microbiological safety, regulatory considerations and the challenges associated with industrial scale-up. By integrating these aspects, this review provides a framework for identifying the conditions under which microbial fermentation can realistically support the development of safe, stable and economically viable functional foods from fruit by-products.

## 2 Fruit waste and non-marketable fruits

Fruit is a key component of a balanced diet, and due to increasing awareness of healthy eating habits, there is a growing demand for this type of food, which also leads to increased waste (Nirmal et al., 2023). Fruit losses occur at all stages of the supply chain because fruit is highly susceptible to mechanical damage, temperature changes, over-ripening, and microbial spoilage (Gonçalves et al., 2025; He et al., 2025; Ul Hasan et al., 2024). Furthermore, industrial fruit processing generates significant quantities of by-products, the type and amount of which depend on the fruit and the processing method. Commonly generated by-products include peels, seeds, pulp, pomace, and bagasse, especially in the production of juices and jams (Gonçalves et al., 2025; Del Rio Osorio et al., 2021).

A substantial proportion of fruit waste consists of produce rejected for cosmetic reasons, such as irregular size or shape, surface blemishes or packaging damage, despite remaining edible and nutritionally valuable. These fruits are commonly discarded because they fail to meet commercial quality standards or consumer expectations regarding appearance. It has been estimated that recovering this underutilised biomass stream could prevent approximately 266,000 tonnes of food waste annually (Hoang et al., 2026). However, despite its considerable valorisation potential, suboptimal fruit has received relatively little attention compared with processing by-products, representing an important opportunity for the development of sustainable bioprocesses and high-value products within a circular bioeconomy framework (Zamani et al., 2026).

In general, the diversity of fruit processing by-products makes them promising substrates for the recovery of bioactive compounds and their application in fermentation processes. Their valorisation not only improves the functional and nutritional properties of derived products but also contributes to enhancing the sustainability and economic efficiency of the fruit processing sector. Table 1 presents a description of the main fruit by-products, their bioactive components and their possible applications.

**Table 1:** Overview of fruit processing by-products, associated bioactive compounds, and valorisation pathways.

By-product	Main components	Potential applications	Key references
**Peels**	Phenolic compounds (flavonoids, phenolic acids), dietary fibre, cellulose, hemicellulose, terpenes, vitamins (A, C, E), minerals	Functional food ingredients, antioxidants, dietary fibre enrichment, cosmetics, pharmaceuticals, bio-based materials (nanocellulose), animal feed	(Leyva-López et al., 2020; Lucarini et al., 2021; Vallejo et al., 2021)
**Seeds**	Lipids (oleic, linoleic acids), proteins, tocopherols (vitamin E), polyphenols, minerals	Edible oils, nutraceuticals, protein supplements, antioxidants, cosmetics	(Gonçalves et al., 2025), (Patra et al., 2022), (Zhang et al., 2024)
**Pomace (pulp + skins + seeds after juice extraction)**	Dietary fibre, polyphenols (anthocyanins, flavan-3-ols, phenolic acids), pectins, minerals	Functional foods, fermented products, dietary fibre fortification, prebiotics, bakery and beverage enrichment	(Leyva-López et al., 2020), (Lucarini et al., 2021), (Gullón et al., 2013)
**Bagasse** (fibrous residue after pulping/pressing)	Cellulose, hemicellulose, lignin, residual sugars, phenolics	Bioethanol, biopolymers, compost, fibre enrichment, fermentation substrates	(Hoang et al., 2026), (Leong and Chang, 2022)
**Pulp residues**	Pectins, soluble fibre, sugars, micronutrients, polyphenols	Thickening agents, functional beverages, probiotic/fermented matrices	(Dranca et al., 2019), (van Trijp et al., 2020)

However, the composition of these substrates also determines their suitability for microbial fermentation and influences microbial growth, metabolic activity, and the extent of subsequent biochemical transformations. The availability of fermentable sugars, organic acids, phenolic compounds, and structural polysaccharides can therefore shape microbial selection and metabolic pathways during fermentation. Understanding these interactions is essential to explain how substrate characteristics are translated into specific biochemical transformations and, ultimately, into changes in product functionality.

## 3 Microorganisms and microbial interactions in fruit-based fermentations

**Fig. 1:**
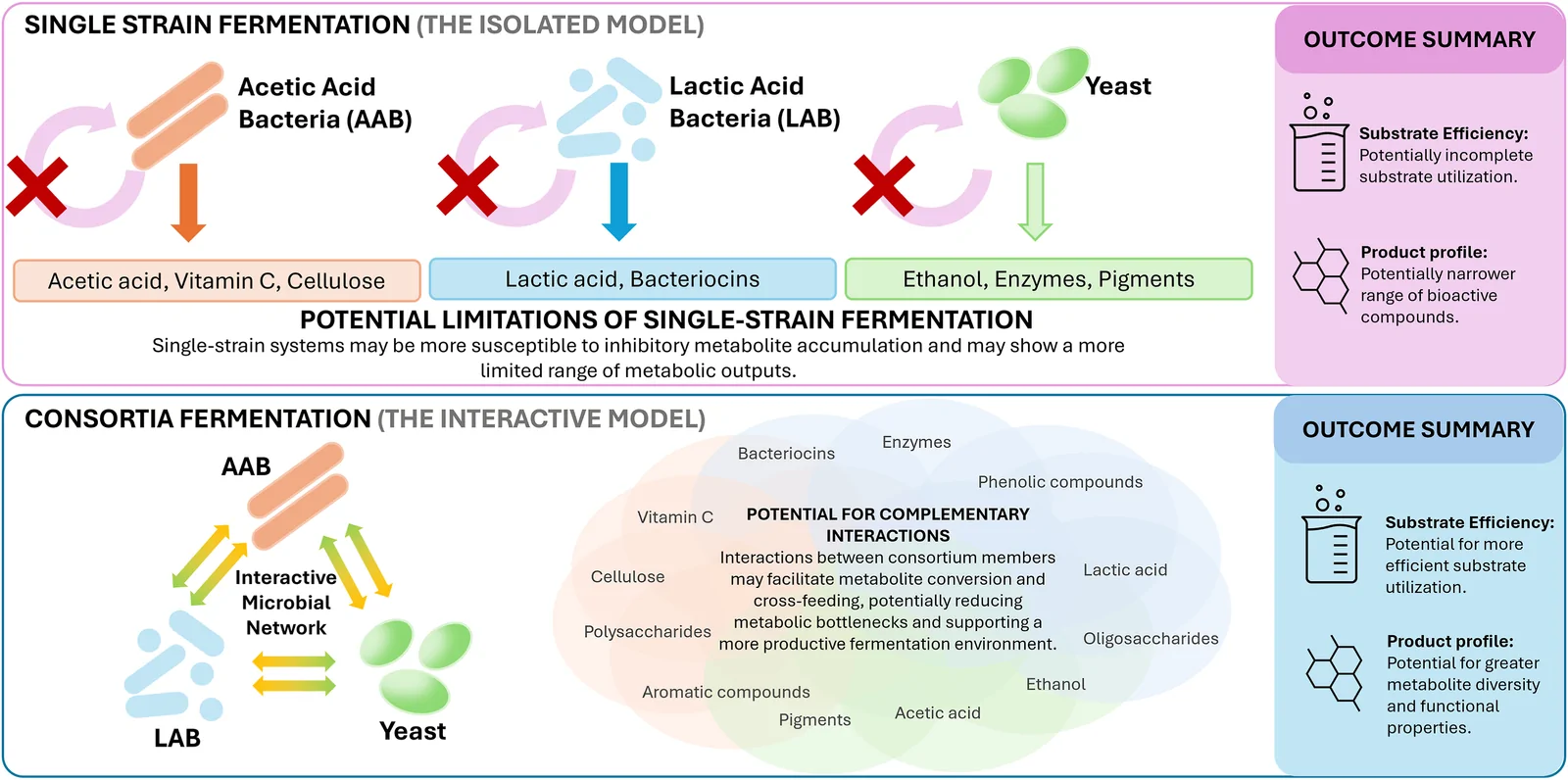
Simplified representation of the possible fermentation processes from individual strains or a consortium.

Fermented fruit products are increasingly recognized as non-dairy functional foods, whose quality and bioactivity are shaped by the interaction between substrate characteristics, microbial metabolism, and fermentation conditions (Calvete-Torre et al., 2023; Tang et al., 2022). The composition of the raw material influences which microorganisms can grow and how they interact and metabolically respond during fermentation, thereby contributing to the diversity of biochemical transformations and metabolites generated. Their safety and reproducibility are also influenced by the composition of the raw material and the microorganisms present at the beginning of fermentation. Many studies have focused on individual species, but in most traditional fermentations, microorganisms are present in complex communities (Fig. 1). Fruit and fruit by-products also carry a diverse native microbiota originating from the fruit surface, soil, water, harvesting equipment, processing environments, and storage conditions (Olaimat and Holley, 2012; Tournas and Katsoudas, 2005; Barth et al., 2009). This microbiota may contribute positively to spontaneous fermentation, but it can also introduce substantial variability and microbiological risks. Therefore, understanding both individual microbial characteristics and community-level interactions is essential for explaining how the characteristics of fruit substrates are translated into specific fermentation outcomes and, ultimately, into the functional properties of the resulting products.

### 3.1 Lactic acid bacteria

LAB represent a diverse group of bacteria, including genera such as *Lactobacillus, Lactococcus*, *Pediococcus*, *Enterococcus*, *Streptococcus*, *Leuconostoc*, and *Weissella* (Zamani et al., 2026). The potential health benefits attributed to them have traditionally been linked to their ability to survive transit along the gastrointestinal tract and their interaction with the host, where they can exert beneficial effects directly or through the metabolites they produce (Husain et al., 2023).

The metabolic capabilities of LAB are very diverse. The most common species exhibit proteolytic, lipolytic, and amylolytic activities, together with the ability to produce lactic acid, a metabolite with important roles in food preservation and industrial applications (Ngouénam et al., 2021). LAB have been isolated from various fruits; for example, *Lactiplantibacillus plantarum* has been isolated from tomato, pineapple, plum, kiwi, papaya, grape, strawberry, and cherries, while *Leuconostoc mesenteroides* subsp. *mesenteroides* and *Pediococcus pentosaceus* have been isolated in cherries (Garcia et al., 2016).

Studies have already shown that LAB have been used to ferment fruit waste and by-products to improve their nutritional and functional properties. Chiarini et al. (2024) employed *Lb*. *plantarum*, *Streptococcus thermophilus*, *Lactobacillus delbrueckii* subsp. *bulgaricus*, and *Limosilactobacillus fermentum* to ferment grape pomace, cocoa bean shells, apple pomace, and defatted roasted hazelnut, resulting in beverages with yogurt-like characteristics. Similarly, Li et al. (2021) demonstrated that LAB strains derived from blueberry (*Lb. plantarum* and *Lb. fermentum*) increased the antioxidant capacity and phenolic content of fermented blueberry products.

However, these properties cannot necessarily be generalized at the species level. Fermentation performance, acidification rate, tolerance to low pH, utilization of fruit-derived carbohydrates, production of antimicrobial compounds, and transformation of phenolic compounds can differ substantially between strains of the same species (Filannino et al., 2015; Cele et al., 2022). Consequently, strain selection should be based on direct characterization in the intended fruit substrate rather than solely on taxonomic identity or performance in conventional laboratory media.

### 3.2 Acetic acid bacteria

Acetic acid bacteria (AAB) are microorganisms with food applications, particularly in vinegar production, vitamin C biosynthesis, and the synthesis of cellulose (De Roos and De Vuyst, 2018). Their metabolism is characterized by the oxidative conversion of sugars and alcohols into organic acids, with the oxidation of ethanol to acetic acid being the most relevant pathway (Fig. 1) (Sengun and Karabiyikli, 2011).

Fruit-based vinegars have recently garnered increasing interest as functional foods due to their content of bioactive compounds. Since AAB are naturally found on the surface of fruits, they are ideal for fermenting fruit substrates and by-products, thus supporting their use in sustainable strategies for valorising fruit (Boondaeng et al., 2022; Dias et al., 2016). Nevertheless, the abundance and composition of native AAB communities can vary according to fruit type, maturity, geographical origin, storage conditions, and processing history. Their activity is also highly dependent on oxygen availability, which is an important consideration when transferring fermentation processes from laboratory flasks to larger industrial vessels (De Roos and De Vuyst, 2018; Gomes et al., 2018).

In many fermented beverages, AAB are found as part of complex microbial communities. In kombucha, AAB along with yeasts and LAB form a structured system consisting of a liquid phase and a cellulose-based biofilm produced primarily by AAB. Within this consortium, microbial interactions and pH reduction contribute to fermentation stability and microbial safety (Fig. 1) (Tran et al., 2020). Dominant genera typically include *Komagataeibacter* and *Gluconobacter*, although the substrate influences community composition, as demonstrated by the prevalence of *Acetobacter* and *Komagataeibacter* in kombucha produced from pineapple waste (Phung et al., 2023; Hata et al., 2023).

Similarly, AABs are found as part of the microbiota of water kefir. They exert their greatest effect in the later stages of fermentation and contribute to the production of organic acids. In water kefir produced from fruit by-products, the dominant genera are *Acetobacter* and *Gluconobacter.* For example, in fermentations of apples, raisins, and figs, the dominant species was *Acetobacter fabarum* (Hata et al., 2023; Zannini et al., 2023).

At industrial scale, the metabolic activity of AAB must be carefully controlled. Insufficient oxygen transfer may limit acetic acid production, whereas excessive aeration can increase evaporation, alter volatile profiles, and promote over-acidification. Moreover, cellulose production by some AAB may be desirable in products such as kombucha but problematic in other processes because it can promote biofilm formation and complicate mixing, cleaning, and downstream processing (De Roos and De Vuyst, 2018; Gomes et al., 2018).

### 3.3 Yeast

Yeasts are central to the natural decomposition of ripe fruits and play a key role in many fermentation processes. In addition to bioactive compounds, fruits contain large quantities of sugars, which are used by yeasts to produce alcohol and other bioactive compounds, such as enzymes, pigments, and antimicrobial compounds (Fig. 1) (Maicas, 2020).

Historically, yeasts have been widely used in fermentation processes for nutritional, medicinal, and industrial applications (Sabater et al., 2020). Regarding the valorisation of fruit waste, given the high efficiency of yeasts in converting sugars into alcohol, bioethanol production has been the most researched approach (Mtashobya et al., 2025; Mgeni et al., 2024; Jahanbakhshi and Salehi, 2019). However, current research is shifting towards their use in the development of fermented beverages and other value-added products.

As previously explained, yeasts play a key role in the production of kombucha and water kefir, but they have also been used to produce various fruit-based fermented beverages. For example, De Matos et al. (De Matos et al., 2017) used a *Pichia kluyveri* isolate obtained from banana waste to ferment the same substrate, producing a brandy with a higher concentration of volatile compounds compared to commercial products. Similarly, pineapple residues have been investigated as a fermentation substrate, with different yeast concentrations leading to beverages with distinct alcohol and sugar profiles (Tran et al., 2022).

As with LAB, yeast performance is highly strain dependent. Strains may differ in sugar utilization, ethanol tolerance, aroma production, stress resistance, and their ability to degrade or transform fruit-associated compounds (Romano et al., 2022). Some yeasts may improve flavour complexity, whereas others may produce undesirable levels of ethanol, off-flavours, or spoilage-associated metabolites. Therefore, the selection of yeast strains for non-alcoholic or low-alcohol fruit fermentations must consider both their technological benefits and their potential to produce ethanol during processing and storage.

### 3.4 Microbial communities

Traditional fermented foods, such as wine, kimchi, and miso, are obtained through the fermentation by microbial communities composed of different microorganisms. During fermentation, complex interactions occur between the microorganisms that cannot be achieved with only a single species; this results in new compounds and enhances functional properties (Fig. 1). Advances in genomic and metagenomic sequencing technologies have allowed for the study of the microbial diversity of traditional fermented foods. This advance has revealed a diversity of microbes exceeding previous analyses with culture-based approaches, revealing other types of microorganisms, which have not yet been fully isolated or characterized, and are also important in the fermentation process (Ponomarova et al., 2017).

In addition to taxonomically identifying microorganisms, sequencing has allowed researchers to investigate microbial metabolism, through which they interact with and modify their environment. This is important for understanding the stability and functionality of traditional fermented foods, how cross-feeding occurs, and coevolutionary processes (Vu et al., 2009). These interactions allow microorganisms to take advantage of complementary metabolic capabilities, improving substrate utilization, metabolite diversity, and the overall robustness of the process.

However, one of the main challenges in designing novel or synthetic microbial consortia for food fermentation is the short adaptation time of the microorganisms to sharing the same space. Unlike traditional fermented products, where microbial communities have coevolved over decades or centuries, newly designed consortia may have fewer opportunities to develop stable interspecific interactions, which could affect their functional performance (Wolfe and Dutton, 2015; Smid and Lacroix, 2013). Therefore, it is important to consider which microorganisms are selected for the consortia based on cross-feeding mechanisms and bacteriocin production. Microorganisms selected for synthetic consortia should also be evaluated both individually and in combination, with particular attention to competition for nutrients, acid tolerance, growth rate, and compatibility under the intended processing conditions (Di Cagno et al., 2013; Leroy and De Vuyst, 2004). The main microbial groups involved in fruit-derived fermentations display complementary metabolic activities that influence acidification, flavour development, bioactive compound transformation and process stability. An overview of their representative genera, metabolic functions, functional contributions and current limitations is provided in Table 2.

**Table 2:** Main microbial groups involved in the fermentation of fruit-derived substrates and their associated metabolic functions, functional contributions, and challenges for application.

Microbial group	Representative genera	Representative substrates/applications	Main metabolic activities	Functional contribution	Main limitations
LAB (Garcia et al., 2016; Chiarini et al., 2024; Filannino et al., 2015; Cele et al., 2022)	*Lactiplantibacillus*, *Levilactobacillus*, *Limosilactobacillus*, *Pediococcus*	Apple pomace, grape pomace, berry juices, citrus by-products	Lactic acid production, phenolic biotransformation, exopolysaccharide production, peptide release	Acidification and preservation, modulation of phenolic profile, improved antioxidant potential, flavour and texture development	Strong strain-dependent variability; limited survival in some harsh plant matrices
AAB (De Roos and De Vuyst, 2018; Gomes et al., 2018; Hata et al., 2023)	*Komagataeibacter*, *Acetobacter*, *Gluconobacter*	Fruit juices, kombucha, water kefir and vinegar-like fermentations	Oxidation of ethanol to acetic acid and other organic acids; cellulose production by selected strains	Acidification, flavour development, microbial stability, formation of cellulose-based matrices	Strict oxygen requirements; sensitivity to process conditions and excessive acidity
Yeasts (Maicas, 2020; Romano et al., 2022)	*Saccharomyces*, *Pichia*, *Hanseniaspora*	Fruit juices, fruit pomaces and mixed fermentations	Sugar conversion to ethanol, CO_2_ and volatile aroma compounds	Aroma complexity, ethanol production, release of flavour compounds, contribution to microbial interactions	Risk of excessive ethanol production; variable technological performance among strains (Maicas, 2020; Romano et al., 2022)
Mixed consortia (Zannini et al., 2023; Ponomarova et al., 2017; Wolfe and Dutton, 2015; Smid and Lacroix, 2013)	LAB + AAB + yeasts	Water kefir, kombucha, fruit-based fermented beverages	Metabolic cross-feeding, sequential substrate utilization and complementary metabolite production	Increased metabolic diversity, complex flavour profiles, potential process robustness	Difficult control of community composition, reproducibility and scale-up

Despite growing interest in this field, current knowledge about metabolic dependencies between species remains relatively limited. Even less is known about the cellular and regulatory mechanisms that govern metabolic exchange and coordination within complex microbial consortia (Sabater et al., 2020). In addition, community composition may change during scale-up, repeated propagation, preservation, or prolonged storage, potentially resulting in the loss of important strains or the dominance of microorganisms with faster growth rates but less desirable functional characteristics (Champagne et al., 2011; Peighambardoust et al., 2011). These interactions and shifts in community composition can directly influence the metabolic activities of the consortium and, consequently, the biochemical transformations occurring in the fermented substrate. Addressing these knowledge gaps will be crucial for the successful development of stable, functional, and safe microbial consortia adapted to the fermentation and valorisation of fruit-derived substrates.

## 4 Microbial and biochemical transformations shaping product functionality

### 4.1 Microbial modification of plant matrices

Fermentation enhances the functionality of fruit-derived substrates through several complementary mechanisms involving microbial metabolism, enzymatic transformation of plant matrices, production of bioactive metabolites and modulation of the physicochemical environment. These mechanisms include microbial succession, acidification, enzymatic degradation of structural components and the release or transformation of bioactive compounds. Although they have been most extensively characterised in traditional vegetable fermentations such as sauerkraut, the same biological principles are increasingly being reported in fruit waste and fruit-processing by-products.

Sauerkraut provides a well-characterised model for understanding these mechanisms. It is produced by fermenting shredded cabbage submerged in approximately 2% brine, where fermentation proceeds through a characteristic succession of lactic acid bacteria (LAB) (Di Cagno et al., 2013; Fukuba et al., 2026). During the initial stages, heterofermentative species such as *Leuconostoc mesenteroides* and members of the genus *Weissella* predominate, producing lactic acid, acetic acid, ethanol, carbon dioxide and other metabolites (Zabat et al., 2018; Tamang et al., 2014; Stamer et al., 1971). Together with the exclusion of oxygen achieved by submergence in brine, acidification, carbon dioxide accumulation and the progressive decline in redox potential create an environment that suppresses many spoilage and pathogenic microorganisms. As fermentation proceeds and the pH decreases, more acid-tolerant LAB, including *Levilactobacillus brevis*, *Pediococcus pentosaceus* and *Lactiplantibacillus plantarum*, become dominant, contributing to fermentation stability and the completion of the process (Stamer et al., 1971). Comparable microbial successions and physicochemical changes have also been described in the fermentation of other plant materials, including cucumbers, carrots and, increasingly, fruit-derived substrates (Smid and Lacroix, 2013).

The progressive acidification and reduction in redox potential not only improve microbial stability but also influence the physicochemical characteristics of the fermented matrix. The acidic and increasingly anaerobic environment contributes to the preservation of sensitive nutrients and limits oxidative deterioration during fermentation and storage. In sauerkraut, for example, these conditions contribute to the partial preservation of vitamin C, which would otherwise be susceptible to degradation during prolonged or inappropriate storage (Siddeeg et al., 2022; Peñas et al., 2010). Similar mechanisms are expected to occur during the fermentation of fruit-derived substrates, although the magnitude of these effects depends on the characteristics of the raw material, the microbial community and the fermentation conditions.

### 4.2 Enzymatic transformation of bioactive compounds

While the physicochemical modifications described above create favourable conditions for fermentation, many of the functional improvements associated with fermented plant materials are due to the enzymatic activity of microorganisms. Depending on the microbial species and strain, and the plant components, these enzymes catalyse the degradation of structural polysaccharides, the hydrolysis of glycosidic bonds, the modification of phenolic compounds, and the proteolysis of plant proteins. Consequently, fermentation not only releases bioactive compounds trapped in the plant matrix but also generates new metabolites with altered bioaccessibility and biological properties (Di Cagno et al., 2013; Filannino et al., 2018).

Plant cell walls are composed primarily of cellulose, hemicellulose, pectin, and lignin, which together form a complex lignocellulosic network that limits the accessibility of intracellular nutrients and bioactive compounds (Di Cagno et al., 2013). During fermentation, microorganisms produce carbohydrate-active enzymes capable of partially degrading these structural polymers, thus increasing substrate accessibility and facilitating the release of sugars, phenolic compounds, and other phytochemicals.

Pectinases are among the most important enzymes involved in the biotransformation of fruit matrices, since pectin is a major structural polysaccharide in the cell walls of fruits and vegetables. While filamentous fungi, particularly *Aspergillus* species, are the main industrial producers of pectinases, pectinolytic activity has also been described in various yeasts and bacteria. Pectin hydrolysis contributes to improved juice extraction and clarification, reduces viscosity, and promotes the release of phenolic compounds. Furthermore, pectin degradation generates pectin-derived oligosaccharides, which have garnered interest as potential prebiotic compounds, since they can subsequently be fermented by the gut microbiota to produce short-chain fatty acids (Haile and Ayele, 2022).

Cellulases and hemicellulases further contribute to the degradation of plant cell wall polysaccharides by disrupting the cellulose-hemicellulose network, thereby enhancing antioxidant recovery and facilitating the extraction of valuable compounds from fruit by-products. These enzymes improve maceration, texture, and the recovery of bioactive components, making them particularly relevant for the valorisation of fruit waste through fermentation (Kuhad et al., 2011).

In addition to the structural degradation of polysaccharides, microbial β-glucosidases catalyse the hydrolysis of glycosidic bonds present in numerous plant secondary metabolites. This reaction releases aglycones from their glycosylated precursors, often increasing their bioaccessibility and simultaneously modifying flavour and aroma profiles by releasing volatile compounds and reducing undesirable bitter compounds (Muradova et al., 2023).

### 4.3 Production of microbial metabolites

In addition to enzymatically modifying plant matrices, fermenting microorganisms synthesise a wide range of metabolites that contribute to the technological, sensory and potential functional properties of fermented fruit products (He et al., 2025). The type and concentration of these metabolites depend on the microbial community, substrate composition and fermentation conditions. Among the most relevant are organic acids, exopolysaccharides, bacteriocins and other bioactive compounds that influence product stability, quality and potential functionality (Mukherjee et al., 2024).

Organic acids are the predominant metabolites generated during fermentation. Lactic acid, produced mainly by lactic acid bacteria, lowers the pH and creates an environment that inhibits spoilage and pathogenic microorganisms while contributing to flavour development and product preservation. In mixed fermentations, acetic acid bacteria oxidise ethanol produced by yeasts into acetic acid, further contributing to acidity and shaping the sensory characteristics of the final product. Besides their technological role, fermentation-derived organic acids may also participate in host–microbiota interactions after consumption. For example, lactate can serve as a substrate for lactate-utilising intestinal bacteria, which convert it into short-chain fatty acids such as butyrate and propionate through microbial cross-feeding. However, the physiological significance of this process following consumption of fermented fruit products remains to be established (Louis and Flint, 2017; Ríos-Covián et al., 2016).

Another important group of fermentation-derived metabolites comprises exopolysaccharides (EPS), synthesized by several LAB and other fermentative microorganisms. These extracellular polymers improve technological properties by increasing viscosity, enhancing water-holding capacity and contributing to product texture and mouthfeel. Depending on their chemical structure, some EPS have also demonstrated prebiotic or immunomodulatory potential in *in vitro* and animal studies, although these biological effects are polymer-specific and require validation in individual food matrices and human studies (Ríos-Covián et al., 2016; Guo et al., 2025).

Fermenting microorganisms also produce antimicrobial metabolites that contribute to product preservation and microbiological stability. In addition to organic acid-mediated acidification, several LAB synthesise bacteriocins, hydrogen peroxide and other antimicrobial peptides capable of inhibiting competing microorganisms (Garcia et al., 2016; Smid and Lacroix, 2013; Di Cagno et al., 2013; Leroy and De Vuyst, 2004). The combined action of these metabolites enhances fermentation stability, extends shelf life and contributes to the microbiological safety of fermented products (Garcia et al., 2016; Smid and Lacroix, 2013).

Beyond these major metabolites, microbial metabolism can generate a variety of secondary compounds, including bioactive peptides, volatile aroma compounds, vitamins and other low-molecular-weight metabolites that contribute to the sensory and nutritional characteristics of fermented foods. The production of these compounds is highly strain-dependent and strongly influenced by substrate composition and fermentation conditions. Consequently, careful selection of microbial consortia represents an important strategy for tailoring the technological and functional properties of fermented fruit-derived products.

### 4.4 Evidence from fruit waste fermentations

Fruit wastes differ markedly in their sugar composition, acidity, fibre structure, phenolic profile, and native microbiota. Consequently, the biochemical changes induced by fermentation are highly dependent on the substrate and strain, making direct comparisons between studies difficult.

One of the most consistent findings is the enhancement of phenolic composition through microbial biotransformation. Sequential fermentations with yeasts and acetic acid bacteria have been shown to generate new phenolic compounds while simultaneously increasing the concentration of organic acids. For example, Yasmin et al. (2025) demonstrated that the sequential fermentation of coconut processing waste promoted the formation of phenolic metabolites absent in the unfermented substrate, illustrating how microbial interactions can amplify the metabolic outcome of fermentation.

Similar improvements have been reported in fermentations of fruit by-products with lactic acid bacteria (LAB). Apple and blueberry waste fermented with *Lacticaseibacillus rhamnosus*, *Lacticaseibacillus casei*, and related species showed higher concentrations of phenolic aglycones after hydrolysis of glycosylated flavonoids, along with improvements in antioxidant capacity (Nawaz et al., 2025; Cheng et al., 2020; Liu et al., 2021; Samarakoon and Rupasinghe, 2025). These observations are consistent with the enzymatic activities described in previous sections, although relatively few studies have directly linked specific enzymes or metabolic pathways to the observed biochemical changes.

Yeasts contribute to fermentation beyond ethanol production by generating volatile aromatic compounds and interacting metabolically with LAB and acetic acid bacteria. In mixed fermentations of grape pomace, the combined activity of yeasts and LAB has also been associated with the formation of metabolites such as gallic acid, pyrogallol, scopoletin, and catechol, which were not detected in the unfermented material. The emergence of these compounds was accompanied by an increase in antioxidant capacity, highlighting the potential of microbial interactions to enhance the functional properties of fermented fruit by-products (Samarakoon and Rupasinghe, 2025).

Despite these promising findings, current evidence remains fragmented. Most published studies evaluate a single substrate–microorganism combination under laboratory conditions using different analytical methods, making cross-study comparisons difficult. Moreover, factors such as microbial viability during processing and storage can strongly influence the stability and functionality of fermented products (Tripathi and Giri, 2014). Nevertheless, the available evidence indicates that the effects of fermentation on product functionality and technological properties can be linked to a limited number of recurring microbial and biochemical mechanisms (Table 3). Future research should focus on mechanistic studies that integrate multi-omics approaches, targeted metabolomics, and controlled fermentation experiments, while also addressing how these transformations can be consistently achieved and maintained during process scale-up and product development.

**Table 3:** Main biochemical mechanisms involved in the functional improvement of fruit-derived substrates during microbial fermentation.

Microbial mechanism	Main microorganisms	Main enzymes/metabolites	Biochemical modification	Potential outcome	Representative examples
Plant cell wall degradation (Dranca et al., 2019; Gullón et al., 2013; van Trijp et al., 2020; Chiarini et al., 2024)	LAB, yeasts, fungi	Pectinases, cellulases, hemicellulases	Breakdown of pectin, cellulose and hemicellulose	Release of phenolics and sugars; improved extraction	Apple pomace, grape pomace
Phenolic biotransformation (Li et al., 2024; Filannino et al., 2015; Cele et al., 2022)	LAB, yeasts	β-glucosidases, esterases, decarboxylases	Glycoside hydrolysis; aglycone formation; phenolic derivatives	Increased bioaccessibility and antioxidant capacity	Blueberry, apple residues
Organic acid production (Garcia et al., 2016; De Roos and De Vuyst, 2018; Gomes et al., 2018)	LAB, AAB	Lactic acid, acetic acid	Acidification	Preservation, flavour development, inhibition of spoilage microorganisms	Most fruit fermentations
Exopolysaccharide synthesis (Vu et al., 2009; Smid and Lacroix, 2013)	LAB	EPS	Polymer synthesis	Improved texture and possible functional properties	Fruit beverages
Aroma generation (Maicas, 2020; Romano et al., 2022)	Yeasts	Alcohol acetyltransferases, glycosidases, esterases	Formation of esters and volatile compounds	Improved sensory quality	Banana, pineapple
Mixed-culture interactions (Zannini et al., 2023; Ponomarova et al., 2017; Wolfe and Dutton, 2015; Smid and Lacroix, 2013)	LAB + yeasts + AAB	Cross-feeding pathways	Sequential metabolite conversion	Greater metabolite diversity and stability	Coconut waste, grape pomace

## 5 Microbial viability and stability in the final product

In fermented plant products, microbial viability has been considered a determining factor of functionality, especially in relation to potential probiotic activity. The presence of microorganisms in the final product depends on several factors, such as substrate composition, fermentation time, temperature, oxygen availability, processing stages, and storage conditions. These parameters significantly influence microbial survival, metabolic activity, and, consequently, the stability and shelf life of the product (Tripathi and Giri, 2014; Ranadheera et al., 2010).

As discussed in previous sections, microbial metabolism drives a range of biochemical transformations in the plant matrix, which can contribute to flavour development, microbial stability, and product acidification. The low pH can protect compounds such as proteins and phenols from degradation (Yang et al., 2024), but it can affect the viability of microorganisms during storage due to the low buffering capacity of fruit matrices (Tripathi and Giri, 2014; Ranadheera et al., 2010).

To address the instability of fermented products, the inactivation or elimination of live microorganisms can prevent secondary fermentation, excessive gas or alcohol increase, and undesirable flavour changes during storage. This process can be achieved through filtration or microbial inactivation via pasteurization. These processes might not interfere with the functional properties of fermented foods, as they can retain their biological activity through microbial metabolites and postbiotics (Salminen et al., 2021; Mukherjee et al., 2024; Marco et al., 2021). These components can include short-chain fatty acids, peptides, exopolysaccharides, cell wall fragments, and transformed phenolic compounds. Experimental evidence, primarily from *in vitro* studies and animal models, suggests that these compounds may exert immunomodulatory, antioxidant and metabolic effects. However, their efficacy in humans depends on the specific postbiotic preparation and remains to be confirmed through well-designed clinical studies (Salminen et al., 2021; Mukherjee et al., 2024).

Yeasts have also been explored as functional agents to enhance the stability and delivery of bioactive compounds derived from fruit processing residues. For example, brewery waste yeast (*Saccharomyces cerevisiae*) has been used as a biological carrier for the encapsulation and protection of phenolic compounds extracted from grape pomace. Spray-dried yeast-based powders exhibit favourable storage properties, including low water activity and reduced hygroscopicity, while improving the bioaccessibility of encapsulated phenolics during simulated gastrointestinal digestion. Although these findings are promising, they are based on *in vitro* digestion models and require validation *in vivo* to confirm their physiological relevance (Rubio et al., 2020).

## 6 Challenges, sustainability, and future prospects

Fermentation of fruit waste offers promising opportunities for the development of functional foods; however, translating the biochemical transformations and functional properties achieved at laboratory scale into safe, stable, and commercially viable products remains complex. The use of heterogeneous substrates, microbial consortia, and emerging processing strategies presents technical, regulatory, and safety challenges that must be carefully addressed. In particular, issues related to microbial functionality, alcohol formation, product stability, consumer acceptance, regulatory classification, and industrial scalability play a decisive role in determining the viability of fermented fruit-based products.

### 6.1 Regulatory considerations for microorganisms and fermented fruit products

The successful commercialisation of fermented fruit products depends not only on technological performance but also on compliance with regulatory requirements governing both the microorganisms employed and the final product. In the European Union, microorganisms included in the Qualified Presumption of Safety (QPS) list are considered safe for their intended use in food applications, whereas in the United States similar evaluations are performed under the Generally Recognized As Safe (GRAS) framework (U.S. Food and Drug Administration; EFSA BIOHAZ Panel, 2020; Marco et al., 2021; Sanders et al., 2019). However, these safety designations should not be interpreted as evidence of probiotic properties or health benefits. According to the current consensus definition, probiotics are live microorganisms that, when administered in adequate amounts, confer a health benefit on the host, and such effects must be demonstrated for the specific strain rather than assumed at the species level (EFSA NDA Panel, 2024; EFSA BIOHAZ Panel, 2017). Consequently, the use of LAB or other microorganisms in fermented fruit products does not justify probiotic or health claims unless these have been scientifically substantiated and comply with the relevant regulatory framework.

In addition to microorganism safety, the regulatory status of the final product must also be considered. Within the European Union, novel foods are defined as foods that were not consumed to a significant degree before 15 May 1997 (EFSA NDA Panel, 2024). Fermented fruit products may fall within this category when they incorporate non-traditional microbial species, novel microbial consortia or genetically modified microorganisms, potentially requiring pre-market authorisation before commercialisation. Furthermore, safety assessments should take into account not only the microbial cultures employed but also characteristics of the final product, including viable microbial counts where relevant, allergenic potential, toxicological considerations and the intended conditions of use. Even when microorganisms possess QPS status, product-specific safety evaluations may still be required depending on the nature of the fermented food and the claims associated with it (EFSA Scientific Committee, 2025).

As interest in fermented fruit-derived foods continues to grow, regulatory frameworks will play an increasingly important role in facilitating innovation while ensuring consumer safety. Early consideration of regulatory requirements during strain selection and product development may therefore accelerate the translation of laboratory-scale research into commercially viable fermented products.

### 6.2 Native microbiota, microbiological risks, and controlled fermentation

Although regulatory approval ensures that selected microorganisms are considered safe for their intended use, the microbiological safety of fermented fruit products also depends on the characteristics of the raw material and the fermentation process itself. Fruit by-products harbour complex native microbial communities that can influence both fermentation performance and product safety.

Fruit surfaces and fruit-processing by-products may support highly diverse microbial populations, including LAB, AAB, yeasts, moulds, and bacteria originating from the environment. The composition of this native microbiota is influenced by fruit species and cultivar, ripeness, climate, agricultural practices, physical damage, post-harvest handling, storage temperature, and processing conditions (Olaimat and Holley, 2012; Tournas and Katsoudas, 2005; Barth et al., 2009). Fruit by-products may present an additional challenge because cutting, pressing, peeling, or pulping can release nutrients and increase the surface area available for microbial growth.

Although the native microbiota may contain microorganisms with useful fermentation properties, it may also include spoilage organisms and potential foodborne pathogens. Depending on the raw material and processing environment, relevant hazards may include members of the Enterobacteriaceae, *Bacillus*, *Clostridium*, *Staphylococcus*, and *Listeria* genera, as well as toxin-producing moulds (Olaimat and Holley, 2012; Tournas and Katsoudas, 2005; Barth et al., 2009). The presence of pesticide residues, mycotoxins, biogenic amines, or other microbial metabolites may also need to be considered. Furthermore, fermentation should not be assumed to eliminate hazards that were already present in the raw material, particularly heat-stable toxins or chemical contaminants (Olaimat and Holley, 2012; Sagar et al., 2018).

Spontaneous fermentation depends on the activity of the native microbiota and may produce desirable products when beneficial microorganisms rapidly become dominant. However, the outcome is difficult to predict because the initial microbial load and community composition can vary considerably between batches. Slow acidification or inconsistent microbial succession may create a period during which spoilage microorganisms or pathogens can persist or grow (Di Cagno et al., 2013; Leroy and De Vuyst, 2004).

Controlled fermentation using selected starter cultures can reduce this variability by promoting rapid microbial dominance and predictable acidification. The resulting reductions in pH, changes in redox potential, nutrient depletion, and production of organic acids, ethanol, carbon dioxide, or bacteriocins can restrict the growth of undesirable microorganisms and improve product stability (Di Cagno et al., 2013; Leroy and De Vuyst, 2004). Nevertheless, safety must be demonstrated for each specific combination of substrate, organism, and process. Appropriate controls may include raw-material screening, washing or pre-treatment, defined inoculum levels, monitoring of pH and temperature, hygienic equipment design, prevention of post-fermentation contamination, and validation of refrigerated or ambient shelf life.

Starter cultures should also be evaluated at the strain level for safety-related characteristics. Relevant assessments include taxonomic confirmation, absence of acquired antimicrobial resistance genes, absence of virulence factors and toxin-production pathways, and compliance with appropriate regulatory frameworks. Microorganisms with qualified presumption of safety or generally recognized as safe status may simplify regulatory development, but this does not remove the need to demonstrate their suitability in the intended food matrix and production process (U.S. Food and Drug Administration; EFSA BIOHAZ Panel, 2020). These considerations highlight that successful fermentation requires not only desirable microbial and biochemical outcomes, but also reproducible and controllable processes that can be reliably implemented beyond laboratory-scale conditions.

### 6.3 Limitations, technological readiness, and industrial translation

#### 6.3.1 Research limitations

Despite promising results, much of the current research on the fermentation of fruit waste and by-products remains at laboratory scale. Many studies use small fermentation volumes, short experimental periods, and tightly controlled conditions that may not adequately represent industrial environments. Differences in substrate composition, inoculum preparation, fermentation time, temperature, oxygen transfer, and analytical methods also make direct comparison between studies difficult (Di Cagno et al., 2013; Sagar et al., 2018).

Fruit by-products are particularly heterogeneous substrates. This variability can influence microbial growth, acidification kinetics, and metabolite production. Some by-products also contain antimicrobial phenolic compounds, essential oils, or other inhibitory substances that may restrict the growth of otherwise suitable starter cultures. Conversely, highly fermentable substrates may support rapid microbial growth but also increase the risk of excessive acid, gas, or alcohol production (Sagar et al., 2018).

#### 6.3.2 Functional evidence

Another important limitation is that many reported benefits are based on a small number of strains or on measurements made immediately after fermentation. Increases in total phenolic content, antioxidant capacity, or other functional indicators do not necessarily demonstrate improved bioavailability or health benefits in humans (Mukherjee et al., 2024; Marco et al., 2021). Similarly, microorganisms detected after fermentation may not remain viable during processing, drying, formulation, storage, or gastrointestinal transit (Champagne et al., 2011). Future studies should therefore distinguish between technological performance, chemical functionality, microbial viability, and demonstrated biological effects. This distinction is also critical for industrial translation, as functional claims must be supported by robust and reproducible evidence before they can be used to justify the development of fermented fruit products.

#### 6.3.3 Methodological limitations and emerging analytical approaches

Methodological limitations also affect the study of microbial communities. Amplicon sequencing can provide information on community composition but generally offers limited strain-level and functional resolution. Shotgun metagenomics can improve taxonomic and functional characterization, but the presence of a gene does not confirm that it is expressed or that the associated metabolic activity occurs during fermentation (Ercolini, 2013; De Filippis et al., 2017; Yap et al., 2022). Metatranscriptomic, metabolomic, and culture-based validation may therefore be required to connect community composition with fermentation performance (Yap et al., 2022). Standardized sampling procedures, sequencing methods, and metadata reporting would also improve reproducibility between studies. Such standardization would also facilitate the comparison of microbial and biochemical outcomes across substrates and fermentation systems, supporting more reliable process development and scale-up.

Current research also tends to focus on a relatively small number of well-characterised microorganisms. While this facilitates comparison between studies and reflects existing safety and regulatory frameworks, it may limit the exploration of functionally promising but less-studied taxa. Broader screening of microbial diversity, combined with appropriate strain-level characterisation and safety assessment, could expand the range of microorganisms available for fruit waste fermentations.

#### 6.3.4 Industrial scale-up

Industrial translation will require greater attention to process robustness and technological readiness. Starter cultures that perform well in small laboratory experiments must also remain stable during biomass production, concentration, freeze-drying or other preservation processes, distribution, and storage (Champagne et al., 2011; Peighambardoust et al., 2011). They must then perform consistently when introduced into variable industrial raw materials.

Scale-up may also alter mixing, heat transfer, oxygen availability, shear stress, and carbon dioxide removal (De Roos and De Vuyst, 2018; García-Ochoa and Gómez, 2009). These changes are particularly relevant for fermentations involving AAB, biofilm-producing organisms, or mixed yeast–bacterial communities. Pilot-scale studies are therefore necessary to establish whether laboratory-scale fermentation kinetics, microbial succession, and metabolite profiles can be reproduced at commercially relevant volumes.

#### 6.3.5 Techno-economic and environmental assessment

Life cycle assessment (LCA) is also required to determine whether fermentation-based valorisation provides a genuine environmental benefit. The use of a waste or by-product as a fermentation substrate should not automatically be considered sustainable, as collection, transport, pre-treatment, sterilization or pasteurization, aeration, agitation, temperature control, concentration, drying, packaging, cold storage, and wastewater treatment may introduce substantial environmental burdens. LCA should therefore compare the proposed fermentation process with realistic alternative uses or disposal routes, such as animal feed, anaerobic digestion, composting, or conventional waste treatment (Lam et al., 2018; Gaffey et al., 2024; Drosou et al., 2023). The functional unit and system boundaries must be clearly defined, and any environmental burdens assigned to the fruit by-product should be transparently reported. Results may differ considerably depending on whether the by-product is treated as burden-free waste or whether part of the environmental impact of primary fruit production is allocated to it. Credits associated with avoided waste treatment and the displacement of conventional products must also be justified (International Organization for Standardization (ISO), 2006; Lam et al., 2018; Gaffey et al., 2024; Drosou et al., 2023). Early-stage assessments should use sensitivity and scenario analyses to address uncertainty in yields, energy use, transport distances, process scale, product substitution, and downstream processing. As the process develops, laboratory estimates should be replaced with primary pilot- or industrial-scale data, and LCA should ideally be performed alongside techno-economic assessment to identify environmental and economic hotspots before commercial implementation (Lam et al., 2018; Gaffey et al., 2024; Drosou et al., 2023).

Further industrial considerations include the cost and availability of substrates, pre-treatment requirements, water and energy use, downstream processing, sensory acceptability, packaging, shelf life, regulatory compliance, and the management of residual solids or liquid waste. The economic value of the fermented product must be sufficient to justify the costs associated with collecting, transporting, and stabilizing fruit by-products, which are often seasonal and geographically dispersed (Sagar et al., 2018; Lam et al., 2018; Gaffey et al., 2024). Taken together, these challenges indicate that industrial feasibility depends on the integration of substrate characteristics, microbial performance, biochemical outcomes, product functionality, and process sustainability rather than on any single parameter.

Future research should therefore progress from proof-of-concept studies towards integrated process development. This should include strain and consortium screening in representative industrial substrates, safety assessment, optimization of inoculum and fermentation conditions, pilot-scale validation, shelf-life studies, sensory analysis, techno-economic evaluation, and LCA (García-Ochoa and Gómez, 2009; Lam et al., 2018; Gaffey et al., 2024; Drosou et al., 2023). Collaboration between microbiologists, food engineers, producers, environmental scientists, and regulatory specialists will be necessary to translate laboratory findings into reproducible, safe, economically viable, and environmentally sustainable fermentation processes for fruit by-product valorisation.

## 7 Conclusions

Fruit waste represents a resource with potential for valorisation within the circular economy. This review highlights that both fruit that does not meet aesthetic standards and the by-products generated after processing possess bioactive compounds that can be recovered, transformed, or improved through microbial fermentation.

Fermentation is a versatile biotechnological strategy capable of converting fruit waste into functional ingredients and foods with enhanced nutritional and technological properties. LAB, AAB, yeasts, and mixed microbial consortia can metabolize complex plant matrices, generating organic acids, transformed phenols, peptides, exopolysaccharides, and other metabolites that contribute to product functionality. It is important to note that these benefits do not depend solely on the presence of viable microorganisms, as postbiotics and fermentation-derived compounds can maintain their biological activity while improving product stability and safety.

The reviewed literature highlights the growing importance of designing microbial communities that enable synergistic interactions, substrate complementarity, and increased metabolic output. However, transitioning to controlled industrial processes remains a challenge, particularly due to substrate heterogeneity, microbial stability, and limited knowledge of metabolic dependencies between species. In addition to scaling up, it is necessary to consider issues related to product stability, including microbial viability after fermentation, alcohol formation, safety, and regulatory compliance. Therefore, particular attention must be paid to microbial safety, reproducibility, storage stability, and economic viability.

In summary, this review provides consolidated evidence supporting the valorisation of fruit waste through fermentation as a promising pathway to reduce food waste, improve resource efficiency, and develop value-added functional foods. Future research should focus on integrating multi-omics approaches, rationally designing microbial consortia, and conducting life cycle assessments to better understand and optimize fermentation-based valorisation strategies. These efforts will be essential to bridge the gap between laboratory-scale research and industrial applications, thereby contributing to more sustainable food systems.

## Author contributions CRediT (contributor roles taxonomy)

•Ines Calvete-Torre: Writing – original draft.

• Samuel Breselge: Writing – review and editing.

• John Leech: Writing – review and editing.

• Harsh Mathur: Writing – review and editing.

• Paul Cotter: Writing – review and editing.

## Funding

This research was supported by Research Ireland under the Government of Ireland Postdoctoral Fellowship Programme, Proposal ID GOIPD/2025/1501.

## Declaration of competing interest

The authors declare that they have no known competing financial interests or personal relationships that could have appeared to influence the work reported in this paper.
